# Correction: Effect of status disclosure on quality of life among people living with HIV/AIDS in Ghana: A health facility-based cross-sectional study

**DOI:** 10.1371/journal.pmen.0000290

**Published:** 2025-03-25

**Authors:** The PLOS Mental Health Staff

## Notice of Republication

This article was republished on 21^st^ February 2025, to correct errors within the author list that were introduced during the typesetting process.

The fifth author’s name is spelled incorrectly. The correct name is: Luchuo Engelbert Bain.

The publisher apologizes for the errors. Please download this article again to view the correct version. The originally published, uncorrected article and the republished, corrected articles are provided here for reference.

## Supporting information

S1 File Originally published, uncorrected article. (PDF)

S2 File Republished, corrected article. (PDF)
