# Supplementary material for: Effect of status disclosure on quality of life among people living with HIV/AIDS in Ghana: A health facility-based cross-sectional study
Source: PLOS Ment Health. 2024 Jun 27;1(1):e0000060. doi: 10.1371/journal.pmen.0000060 (PMC12798609; doi:10.1371/journal.pmen.0000060)
Supplement: Attachment — Submitted filename: Response to Reviewers.docx [file pmen.0000060.s002.docx]

**Reviewer #1:**

*General comments:*

The study investigated an important public health issue, from the perspective of resource-limited setting. However, I have itemized my comments, concerns, and suggestions below.

1. *Title:* The title is clear and concise.

**Response:** Thank you.

2. *Abstract*: The abstract is good summary of the whole manuscript, including the results presented.

**Response:** We appreciate your comment.

3. *Introduction:* The introduction is well written. The research problem and study rationale were clearly stated by the authors.

**Response:** Thank you for your comment.

*Methods and materials*

4. *Study site and population:* The information on the study site appears scanty. The authors should please kindly provide additional information on the study setting as related to HIV/AIDS treatment in particular and healthcare in general.

**Response:** Thank you. We have provided more details describing the study site on page 6.

*Sampling*

5. The sample size calculation does not seem right, especially the population size used for the calculation. This made the sample size required for the study appear rather too small. Using the total number of PLWHA as contained on the register of the HIV clinics selected for the study would have been more appropriate. The small sample size in this study will limit the application of its findings beyond the study group.

**Response:** The hospital is one of three study sites for a planned intervention study (phase two of the bigger study). Indeed, the we used the total number of PLWHA as contained on the register of the ART unit of the selected hospital for the study’s sample size determination. This has been revised on page 8. The focus of the study was to understand the baseline situation at the facilities for targeted intervention.

*Data analysis*

6. Linear regression does not seem appropriate for the dataset. Normality is not the only requirement for running linear regression test. The dependent and independent variables must have a linear relationship. Both dependent and independent variables must be measured at the continuous level. I do not think the variables in this study, especially the independent variables fulfilled these requirements. In the study, the independent variables are categorized and are nominal data. The authors should consider conducting chi-square test followed by binary logistic regression. In doing so, the authors should first classify the health-related quality of life scores into two groups (good & poor).

**Response:** Categorising the QoL as good & poor will shadow the internal variability in the scores. We intended to understand how the independent variables explained the changes in the QoL scores to inform intervention design. As such, we treated the dependent variable as a continuous variable. Prior to running the linear regression, the three main assumptions for using linear regression were confirmed. With regards to the categorical independent variables, we converted into dummy variables to set up the model. This has been explained in the Data analysis section on page 9.

7. *Discussion:* The findings are well discussed in view existing literature. However, the authors should give attention to the concerns raised above.

**Response:** Thank you for your feedback.

8. *Conclusion:* The conclusion is based on the results presented.

**Response:** Thank you.

**Reviewer #2:**

1. This study addresses a pivotal subject matter, focusing on the impact of status disclosure on the quality of life (QoL) of individuals living with HIV/AIDS (PLWHA). However, there is a suggestion that employing a qualitative or case-control study design would have been more advantageous than the chosen cross-sectional approach.

**Response:** Thank you. Your suggestion is duly noted. However, this manuscript reports on the quantitative component of the broader study, a mixed methods study, that set to identify underlying factors and baseline information about PLWHA’s QoL and social support to inform intervention design.

2. In assessing the effects of status disclosure on QoL, how did the authors precisely determine the time elapsed since disclosure (in days, weeks, or months) and categorize participants as "hospitalized" or "outpatients"? Additionally, what methodology was employed to conclude that the specific aspect of "status disclosure" significantly affected participants' quality of life based on events occurring in the past month, utilizing the WHOQOL Bref instrument?

**Response:** While the time elapse since disclosure and even diagnosis is significant to understanding the linkage between status disclosure and QoL, most respondents could not recall the time elapse. The time elapse variable were thus expunge in the final data. There were no hospitalized PLWHA included in the study. As such, we did not have any variable categorizing the participants as “hospitalized” or “outpatient”. The study however did not conclude that “specific aspect of "status disclosure" significantly affected participants’ quality of life”. The study’s premise based on literature was that status disclosure to persons aside one’s healthcare provided avails access to social support that improves the quality of life. This has been stated in the introduction, page 4.

3. The exclusion of seriously ill PLWHA from the study is mentioned. Could the authors provide clarification on the criteria used to classify the status of patients, leading to their exclusion?

**Response:** The criteria used to classify the patients as being seriously ill have been included on page 7 of the manuscript. The criteria comprised: experiencing a high burden of symptoms related to HIV/AIDS, such as pain, fatigue, or cognitive impairment, and significant limitations in ability to perform daily tasks due to HIV/AIDS-related symptoms or complications.

4. The study suggests that qualitative methods are superior for assessing quality of life, yet a cross-sectional survey design was chosen. Can the authors elaborate on the rationale behind this choice, especially considering the acknowledged importance of the subject matter?

**Response:** The study did not suggest that qualitative methods are superior for assessing quality of life. We stated that some factors that may influence QoL such as sociocultural factors can only be assessed qualitatively. We acknowledge that there are some social determinants of QoL which can best be understood in terms of the impact mechanism through qualitative methods. This was not the focus of the current study although it was covered in the second phase of the mother mixed methods study. This has been clarified on page 25.

**Reviewer #3:**

1. The study addresses the crucial topic of HIV/AIDS status disclosure and its potential impact on the quality of life (QoL) among People Living with HIV/AIDS (PLWHA) in Ghana. Given the limited number of studies specifically addressing QoL among PLWHA in Ghana, this study is deemed important. Even with a small and non-representative sample, the study holds value.

**Response:** We appreciate your review and comments

2. POINT -1 The introduction effectively emphasizes the importance of the well-being of individuals with HIV. Nevertheless, the text emphasizes the benefits without sufficiently addressing the complex socio-cultural factors contributing to the quality of life for individuals. I suggest including information about stigma and discrimination, particularly in the context of sub-Saharan Africa and Ghana. In the ethics section, the authors must include the ethics committee approval number.

**Response:** The Introduction section has been revised to include more information about stigma and discrimination in the context of SSA and Ghana on page 5. The ethical approval number has been included on page 10.

3. POINT 2 - The method used is adequate, following the STROBE guide. The statistical method used is suitable for epidemiological studies.

**Response:** Thank you.

4. POINT 3- The study provides valuable insights into the low rate of HIV/AIDS status disclosure among PLWHA in the Obuasi East District, aligning with global trends. While the study identifies several predictors of quality of life (QoL) among PLWHA, it lacks a proposed cause for the non-significant link between disclosure and QoL. I suggest reinforcing the importance of factors influencing QoL, such as supportive relationships and reduced stigma. The discussion presents intriguing findings regarding the impact of age, gender, geographic location, and religion on the QoL of PLWHA. However, the study could benefit from exploring how age, gender, location, and religion may interact to shape the experiences and well-being of PLWHA. Other countries yield results on these sociodemographic characteristics.

**Response:** Importance of factors influencing QoL, such as supportive relationship and reduced stigma has been reinforced to proposed cause for the non-significant link between disclosure and QoL on page 21. How the sociodemographic characteristics interact to shape experiences and well-being for PLWH has been discussed on pages 22-24.

5. POINT 4- In future directions, there is room for a more nuanced discussion regarding the implications of the study's findings for healthcare policies and interventions in Ghana. Further exploration of the factors contributing to the observed disparities in QoL, especially socio-demographic predictors, could offer valuable insights for targeted interventions aimed at improving the well-being of PLWHA in different demographic groups. Additionally, it would be beneficial to highlight the limitations of the study more explicitly, such as the potential biases associated with a cross-sectional design in the specific setting, to enhance the interpretation and generalizability of the findings. It is suggested to reiterate information about the scenario in the realized study and its representation in Ghana.

**Response:** The discussion regarding the implications of the study’s findings for healthcare policies and intervention in Ghana has been expanded throughout the revised discussion (pp. 20-24). Also, the limitations of the study have been highlighted more explicitly to enhance the interpretation and generalizability of the findings (page 25). The revised discussion has reiterated information about the scenario in the study and its representation in Ghana.
