# Supplementary material for: Effect of status disclosure on quality of life among people living with HIV/AIDS in Ghana: A health facility-based cross-sectional study
Source: PLOS Ment Health. 2024 Jun 27;1(1):e0000060. doi: 10.1371/journal.pmen.0000060 (PMC12798609; doi:10.1371/journal.pmen.0000060)
Supplement: Attachment — Submitted filename: Response to Reviewers 2.docx [file pmen.0000060.s003.docx]

**Response to Reviewers’ Comments**

**Journal Requirements:**

Comment 1: Please review your reference list to ensure that it is complete and correct. If you have cited papers that have been retracted, please include the rationale for doing so in the manuscript text, or remove these references and replace them with relevant current references. Any changes to the reference list should be mentioned in the rebuttal letter that accompanies your revised manuscript. If you need to cite a retracted article, indicate the article’s retracted status in the References list and also include a citation and full reference for the retraction notice.

Response: The reference list has been reviewed to ensure that it is complete and correct. None of the references has been retracted.

**Reviewer #4:**

Comment 1: not following Vancouver reference styling in citation (please refer to submission guidelines)

Response: The references have been revised to Vancouver reference styling

Comment 2: English language used is not professional for article research

Response: Revision has been made to address any nonprofessional expressions in the manuscript.

Comment 3: Abstract should be restructure

Response: The abstract follows the expected structure and word-limit.

**Reviewer #5:**

Comment: Good luck

Response: Thank you very much.

**Reviewer #6:**

Comment 1: Please write the full form acronym (eg PEPFAR) when used for the first time in the text.

Response: The full form of the acronym

Comment 2: In the study site and population - please share if written informed consent has been taken from the individuals or not. It is not clear.

Response: Written informed consent was obtained from all the respondents prior to inclusion as already stated in the Ethical Issues section on page 10.

Comment 3: How was the confidentially of the individuals maintained - should be written in detail.

Response: How confidentiality of the respondents was maintained has already been clearly explained in the Ethical Issues section to cover data protection from third-parties and deidentification of the data on page 10

Comment 4: Please REFRAME - Persons accessing ART at the hospital were SELECTED and approached after they had received their service, and the purpose of the study was explained to them. (how can a researcher select without written informed consent and first enrolling individual in the study)

Response: The statement has been clarified.
