# Supplementary material for: Correction: Effect of status disclosure on quality of life among people living with HIV/AIDS in Ghana: A health facility-based cross-sectional study
Source: PLOS Ment Health. 2025 Mar 25;2(3):e0000290. doi: 10.1371/journal.pmen.0000290 (PMC12798332; doi:10.1371/journal.pmen.0000290)
Supplement: S1 File — (PDF) [file pmen.0000290.s001.pdf]

RESEARCH ARTICLE

Effect of status disclosure on quality of life among people living with HIV/AIDS in Ghana: A health facility-based cross-sectional study

Farrukh Ishaque Saah[1,2]*, Kizito Aidam[3], Abdul-Aziz Seidu[1,4], Hubert Amu[5], Luchet Engelbert Bain[6]

1 Department of Population and Health, Faculty of Social Sciences, University of Cape Coast, Cape Coast, Ghana, **2** Public Health Emergency Operations Centre, Clinical and Public Health Services Department, Ministry of Health, Kigali, Rwanda, **3** Department of Family and Community Health, School of Public Health, University of Health and Allied Sciences, Hohoe, Ghana, **4** College of Public Health, Medical and Veterinary Sciences, James Cook University, Townsville, Australia, **5** Department of Population and Behavioural Sciences, School of Public Health, University of Health and Allied Sciences, Hohoe, Ghana, **6** International Programs, African Population and Health Research Center, Nairobi, Kenya

* fsaahpnur14@uhas.edu.gh

Check for updates

OPEN ACCESS

Citation: Saah FI, Aidam K, Seidu A-A, Amu H, Bain LE (2024) Effect of status disclosure on quality of life among people living with HIV/AIDS in Ghana: A health facility-based cross-sectional study. PLOS Ment Health 1(1): e0000060. https://doi.org/10.1371/journal.pmen.0000060

Editor: Isabella Giulia Franzoi, University of Turin, ITALY

Received: December 6, 2023

Accepted: May 6, 2024

Published: June 27, 2024

Peer Review History: PLOS recognizes the benefits of transparency in the peer review process; therefore, we enable the publication of all of the content of peer review and author responses alongside final, published articles. The editorial history of this article is available here: https://doi.org/10.1371/journal.pmen.0000060

Copyright: © 2024 Saah et al. This is an open access article distributed under the terms of the Creative Commons Attribution License, which permits unrestricted use, distribution, and reproduction in any medium, provided the original author and source are credited.

Data Availability Statement: The data has been made available as a supporting file.

Funding: The authors received no specific funding for this work.

Abstract

Status disclosure to family and friends among people living with HIV/AIDS (PLWHA) is necessary to ensure social and material support, which are important predictors of quality of life. We examined HIV/AIDS status disclosure and its influence on quality of life (QoL) among PLWHA in a Ghanaian population. This was a health facility-based cross-sectional study of 124 PLWHA accessing antiretroviral therapy at a hospital in the Ashanti Region of Ghana. Data were collected using a pretested questionnaire adopting the WHO HIV-QOL BREF. The data were analysed descriptively and inferentially using STATA 15.0. Statistical significance was set at a p value<0.05 at a 95% confidence interval. Forty-two percent of the respondents disclosed their HIV status. Social support was available for 76.9% of those who had disclosed their status. The mean QoL was highest within the physical domain (14.3 ±2.9), while the psychological domain indicated the lowest quality of life (11.03±3.26). Approximately 47.6% attained the mean overall QoL score (73.18). Being a male predicted lower QoL in all domains than being a female. Being a Muslim, having tertiary education, and being an urban resident significantly predicted better QoL than being a Christian, having no formal education, and being a rural resident, respectively. Respondents' age negatively predicted overall quality of life. Although disclosing one's HIV/AIDS status positively predicted QoL, the difference was not statistically significant. A significantly poor quality of life among patients can drag efforts by Ghana towards achieving the Sustainable Development Goal of reducing the HIV/AIDS pandemic and its associated mortality. Stakeholders in AIDS prevention and management in Ghana should employ innovative methods such as peer support to encourage status disclosure and provide education on the provision of social and emotional support for PLWHA.

Competing interests: The authors have declared that no competing interests exist.

Introduction

The advent of antiretroviral therapy (ART) and effective treatment protocols have provided opportunities for PLWHA to live longer lives; hence, HIV is now perceived as a manageable chronic condition instead of a terminal illness [1]. Indeed, there is conclusive evidence that effective ART reduces the risk of HIV transmission to almost zero when the viral load remains persistently below 1000 copies per ml [2]. The treatment also significantly improves life expectancy and health-related quality of life [3]. The U.S. President's Emergency Plan for AIDS Relief (PEPFAR) recently estimated that approximately 39 million people are living with HIV/AIDS worldwide, and 29.8 million out of this number have access to antiretroviral treatment [4]. Adopting and scaling up the most effective strategies to prevent new infections has been central to the HIV/AIDS control strategy [5]. This strategy is aimed at improving the quality of life (QoL) of people battling the infection [5].

Self-disclosure of sensitive information is beneficial for people's health: lower stress and better psychological well-being [6, 7]. Studies also indicate that individuals who disclosed their results have greater adherence to ART treatments [8–10]. Moreover, disclosure may increase opportunities to receive social support, which may help individuals cope and recover from physical illness and lessen depressive symptoms due to HIV-related physical symptoms [11, 12].

Folasire and colleagues argue that the availability of care and social support from family members and close friends are major determinants of the QoL of PLWHA [13]. Although willing to access this support and care, most PLWHA are concerned about the possibility that disclosing their HIV status to family members and close friends will result in some stigmatization and discrimination, likely affecting their loved ones and personal relationships [14, 15]. The negative attitude of society and HIV-related stigma dissuade PLWHA from disclosing their status, especially in low- and middle-income countries [16, 17]. Stigma has profound implications for HIV prevention, treatment, care, and support [18, 19].

Studies have shown reluctance to disclose HIV status among many sub-Saharan Africans even when they had gone for Voluntary Counselling and Testing, with the few who choose to disclose it being selective in choosing their audience [20–23]. Stigma and discrimination against PLWHA are pervasive and represent significant obstacles to HIV/AIDS prevention efforts, including HIV testing and status disclosure [24, 25]. Internalised and experienced/expected HIV-related stigma, such as guilt, shame, fear of abuse, and rejection, among other things, is a major obstacle to HIV status disclosure in Ghana and some parts of Sub-Saharan Africa [26–28]. In Ghana, HIV status disclosure remains a difficult task for most HIV-positive individuals poses a real challenge in providing treatment and clinical care to PLWHA [29]. Internalised HIV stigma is common among PLWHA in Ghana while community-related stigma has been found to affect HIV status disclosure [30]. Acquiring HIV/AIDS in Ghana is generally considered the result of promiscuity or sexual immorality, so infected individuals are highly discriminated against and stigmatized [31]. PLWHA are subjected to a variety of types of stigmatisations and discrimination, such as rejection from their families and communities, inability to share a meal or a bed, finger-pointing, and exclusion [25, 32]. These discriminations and stigmatization lead to decreased self-esteem, lowered body image, negative feelings about self, depression, anxiety, self-isolation, feelings of guilt and failure to disclose status [33].

Chronic illness has always constituted a barrier to QoL [34, 35]. HIV/AIDS is notorious for hindering the QoL of people [34]. PLWHA face psychological isolation and condemnation from their family, friends, and society because people around them are aware of their HIV status, affecting their physical health even though ARTs help with regaining their strength [36, 37].

Disclosure to family members and friends is indeed necessary to ensure social and/or material support [38], especially when the availability of care and social support from family members and close friends tend to be the major determinants of the QoL of those living with HIV in resource-limited settings [13]. It is also noted that the desire for care and support enforces the willingness to disclose one's HIV status; however, this is withheld where support is not expected [13].

There is a paucity of information regarding disclosure and QoL among PLWHA in the Obuasi East District of the Ashanti Region. It is therefore of prime importance to know what the effect of HIV status disclosure has been on PLWHA. Therefore, we examined the influence of HIV/AIDS status disclosure on quality of life among PLWHA in the Obuasi East District of the Ashanti Region of Ghana.

Methods and materials

Ethics statement

Ethical approval was obtained from the Research Committee of the District Health Directorate (OMHD/2020/24). Administrative permission was sought from the hospital management. Prior to inclusion in the study, written informed consent was obtained from patients, which involved signing/thumbprinting an information sheet describing and explaining the purpose of the research, benefits, potential harms and confidentiality of respondents to prevent victimization of any form. All research was performed in accordance with relevant guidelines/regulations. The study did not collect any personal identifying information of the respondents; instead, pseudonyms were used to protect the anonymity and privacy of the respondents. The data have been deidentified and stored on the password-protected computer of the authors without access by any third parties to ensure the confidentiality of the respondents.

Study design

This study adopted a health facility-based cross-sectional design. It involved the collection of quantitative data from a section of PLWHA from a health facility at a single point in time. This report followed the Strengthening the Reporting of Observational Studies in Epidemiology (STROBE) guidelines.

Study site and population

The study was carried out at a hospital in the Obuasi East District in the Ashanti Region of Ghana. The district was carved out of the Obuasi Municipality to become one of the 260 districts in Ghana [39]. The capital is Tutuka. The hospital served as one of the three designated study sites for a planned intervention study, representing a pivotal hub for healthcare services in the region. Notably, it was among the earliest facilities to initiate ART services within the district, signifying its crucial role in providing HIV/AIDS treatment and care. As a primary centre for ART services, the hospital plays a vital role in addressing the healthcare needs of individuals living with HIV/AIDS in the local community. This study site was selected due to its significance in delivering comprehensive HIV/AIDS care and its accessibility to a diverse patient population within the district.

The study involved PLWHA who were accessing ART services at a health facility in the Obuasi Municipality. This population was chosen because they are experiencing service that is meant to impact the phenomena of this study, the decision to disclose HIV serostatus and QoL. However, PLWHA who were seriously ill were excluded. This included individuals experiencing a high burden of symptoms related to HIV/AIDS, such as pain, fatigue, or

cognitive impairment, and those with significant limitations in their ability to perform daily tasks due to HIV/AIDS-related symptoms or complications.

Sampling

The hospital's ART clinic has 210 PLWHA registrants who are currently receiving ART services. Hence, the study utilized the sample size calculation formula by Yamane [40] $n = \frac{N}{1+N(e)^2}$, where N is the population size (210) and e is the margin of error at a 95% confidence interval (0.05). The estimated sample size was 138.

The study adopted a simple random sampling technique, a balloting approach. The ART Register was used as the sampling frame, and balloting was performed on the registration numbers. Individuals whose numbers were selected and consented were included in the study. However, where a selected patient refused to consent, they were replaced with other patients from the register who provided consent.

Study variables

The dependent variable in this study was health-related QoL among PLWHA. This variable was determined using the WHOQOL BREF tool [41]. The independent variables included the sociodemographic characteristics of the respondents, such as age, sex, religion, ethnicity, level of education, occupation, duration of infection awareness, and HIV serostatus disclosure. HIV serostatus disclosure in this study referred to disclosure of one's HIV/AIDS status to a third party (e.g family, friends, etc.) from a healthcare service provider.

Procedures

Data were collected using a pretested questionnaire (Cronbach's alpha reliability = 0.804). The questionnaire was sectioned into three parts: sociodemographic information, status disclosure, and quality of life. The WHOQOL-HIV BREF instrument was adopted to assess the respondents' quality of life. The instrument is used to appraise QoL as well as respondents' overall perception of their health using 31 questions evaluating QoL from six domains, namely, physical, psychological, level of independence, social relation, environmental, and spiritual domains. These domains are scored on a 5-point Likert scale with 5-very good and 1-very poor [41]. Persons accessing ART at the hospital were selected and approached after they had received their service, and the purpose of the study was explained to them. Upon completion of the study participation process and assurance of ethical concerns and all other concerns addressed, a person living with HIV who consented to participate in the study was interviewed. Data were collected with the help of three research assistants between October 15 and November 10, 2020. Respondents who could not read or understand the questionnaire were assisted, and the questions were explained to them.

Data analyses

Data (S1 Data) collated were coded and entered into the EpiData version 4.3 template and exported to Stata version 15 (Stata Corporation, College Station, TX, USA) for cleaning and analysis. Descriptive statistics such as frequency, mean, and percentage were generated to summarize the variables. A multiple linear regression analysis was conducted to test the predictors of QoL. QoL is normally distributed. The data was tested to confirm the key assumptions for conducting a linear regression test. Categorical independent variables were converted into dummy variables in preparation to set up the model. The linearity assumption was established to confirm linear relationship between the variables. All the independent variables except

status disclosure were tested for their strength of association and prediction in the initial step. A second multiple linear regression was conducted with all the independent variables inclusive of status disclosure to determine the effects of status disclosure on QoL and its predictors. Statistical significance was considered at a p value of less than 0.05 at a 95% confidence interval. The QoL variable was analysed following the WHO guidelines for analysis of the WHOQOL--HIV BREF tool and grouped into six intermediary variables: physical, psychological, social, environmental, level of independence, and spiritual domains [41]. Total scores of each of the six domains were calculated from the sum of the responses of the domain's items. The overall QoL variable was then computed by summing the total scores of the six domains.

Results

Sociodemographic characteristics of respondents

In all, 124 PLWHA consented to participate, resulting in an 89% response rate. Table 1 shows the sociodemographic characteristics of the respondents. Most of the respondents (54.8%) were males. The mean age of the respondents was 35.38 years. Additionally, 58.0% of the respondents were never married, and 20.2% were divorced/widowed. Most of the respondents (62.1%) were Christians, and 14.5% were Muslims. Furthermore, more than half (72.6%) of the respondents were Akan, and 45.2% had attained secondary education. Almost all (80.6%) of the respondents were urban residents. In addition, more than half (58.9%) were employed. Of those employed, 20.5%, 21.9%, and 19.2% were traders, skilled workers, and artisan miners, respectively.

Status disclosure and social support availability among PLWHA

Regarding status disclosure, 41.9% of the respondents disclosed their status to another person. Of these, a relative majority (46.2%) disclosed to their spouses, and 13.4% and 23.0% disclosed to children and nonfamily relations, respectively. Additionally, 76.9% of those who disclosed their HIV/AIDS status received a form of social support from such persons. Kinds of support received included support for medical expenses (47.5%), cash for upkeep (55.0%), shelter (52.5%), and emotional support (55.0%). The main sources of emotional support were spouses (60.0%), children (17.5%), parents (7.5%), and nonfamily relations (7.5%). Additionally, 16.9% of the respondents were in peer support groups (Table 2).

Quality of life of people living with HIV/AIDS

This study also assessed the QoL of the respondents in Table 3. The mean QoL was highest within the physical domain (13.3±2.9), while the psychological domain indicated the lowest QoL among the respondents (11.03±3.26). The overall mean QoL score was 73.18, with 47.6% attaining at least this score.

Table 4 presents the results of a comparison of the means of QoL per background characteristics of the respondents. On average, respondents who were less than thirty years old had higher QoL in the physical, psychological, independence, social and environmental domains, and the difference was statistically significant. This same age group averaged a higher overall QoL, and the difference was statistically significant. Females recorded higher average QoL scores than males in physical, psychological, and independence domains as well as overall QoL, and the difference was statistically significant. Those who were currently married had higher average QoL scores in all domains, including overall QoL, and the difference was statistically significant. Regarding religion, Muslims had higher average QoL scores in all domains, including overall QoL, and the difference was statistically significant. Respondents with

Table 1. Sociodemographic characteristics of respondents.

Sociodemographic variable	Frequency	Percentage (%)
**Sex**		
Male	68	54.8
Female	56	45.2
**Age (in years)**	**(Mean = 35.38±8.883)**	
<30	32	25.8
30–39	52	41.9
40–49	30	24.2
50+	10	8.1
**Marital status**		
Never married	72	58.0
Married	27	21.8
Divorced/widowed	25	20.2
**Religion**		
Christianity	77	62.1
Islam	18	14.5
None	29	23.4
**Ethnicity**		
Akan	90	72.6
Ewe	9	7.2
Mole-Dagbani	25	20.2
**Educational level**		
No formal education	16	12.9
Primary	21	16.9
Secondary	56	45.2
Tertiary	31	25.0
**Residence**		
Rural	24	19.4
Urban	100	80.6
**Employment status**		
Employed	73	58.9
Unemployed	51	41.1
**Occupation**		
Trader	15	20.5
Farmer/breeder	7	9.6
Skilled work	16	21.9
Teacher	12	16.5
Unskilled labourer	14	19.2
Other	9	12.3
**Years since diagnosis**	**(Mean = 12.35±5.97)**	
<10	40	32.3
10–19	67	54.0
20+	17	13.7

https://doi.org/10.1371/journal.pmen.0000060.t001

tertiary education had a higher average QoL score in all domains, including overall QoL, and the difference was statistically significant. Even though respondents who disclosed their status had higher overall scores for all QoL domains except independence, the difference was not statistically significant.

Table 2. Status disclosure and social support availability.

Variable	Frequency (N = 124)	Percentage (%)
**HIV/AIDS status disclosure**		
Yes	52	42
No	72	58
**Person of first disclosure (n = 52)**		
Spouse alone	24	46.2
Children alone	7	13.4
Spouse and children	3	5.8
Parents	3	5.8
Extended family	3	5.8
Nonfamily relations	12	23.0
**Availability of social support (n = 52)**		
Yes	40	76.9
No	12	23.1
*****Kind of support received (n = 52)**		
Funds for medical expenses	19	47.5
Cash for upkeep	22	55.0
Shelter	21	52.5
Support with food	30	75.0
Support for clothing	18	45.0
Emotional support	22	55.0
**Having a support group**		
Yes	21	16.9
No	103	83.1

*Multiple response

https://doi.org/10.1371/journal.pmen.0000060.t002

Factors influencing quality of life among PLWHA

Table 5 presents the effects of status disclosure and other independent factors on the QoL of PLWHA. The regression model explained 51.5%, 54.0%, 55.6%, 41.2%, 48.7% and 44.4% of the variance in the physical, psychological, independence, social, environmental and spiritual domains, respectively. Overall, the regression model explained 51.8% of the variance in the overall quality of life score. As age increased, overall QoL decreased, and this observation was statistically significant. The QoL of males was significantly lower than that of females in all domains as well as overall QoL. Muslims had significantly higher QoL than Christians in all domains as well as overall QoL. Respondents who had tertiary education had significantly

Table 3. Summary scores of WHOQOL-HIV domains.

QoL dimension	Mean score	Minimum score	Maximum score	95% confidence interval
Physical	13.63	4.00	19.00	12.98–14.28
Psychological	11.03	4.80	17.60	10.45–11.61
Independence	11.94	7.00	16.00	11.34–12.44
Social	12.83	7.00	19.00	12.29–13.37
Environmental	11.38	8.00	16.00	11.09–11.72
Spiritual	12.38	9.00	19.00	11.95–12.81
Overall QoL	73.18	43.30	101.80	70.51–75.84

https://doi.org/10.1371/journal.pmen.0000060.t003

Table 4. Comparison of mean QoL scores for background characteristics.

Variable	QoL domains						
	Physical	Psychological	Independence	Social	Environmental	Spiritual	Overall QoL
	Mean(S.D.)	Mean(S.D.)	Mean(S.D.)	Mean(S.D.)	Mean(S.D.)	Mean(S.D.)	Mean(S.D.)
**Age group**							
<30	16.03(2.33)	12.70(2.32)	14.06(1.54)	14.44(2.65)	11.94(1.42)	12.22(1.58)	81.39(8.87)
30–39	13.12(3.22)	10.65(3.03)	11.81(2.60)	12.08(2.56)	11.03(1.95)	12.33(2.53)	71.00(13.89)
40–49	13.20(4.50)	10.88(4.06)	11.20(2.87)	13.50(3.38)	11.85(2.31)	12.90(3.20)	73.53(18.90)
50+	9.90(1.85)	8.08(1.16)	8.00(0.82)	9.60(1.71)	9.95(1.09)	11.60(0.52)	57.13(2.62)
P value	<0.001	<0.001	<0.001	<0.001	<0.01	0.462	<0.001
**Sex**							
Female	17.71(2.96)	11.79(3.01)	13.00(2.39)	13.41(2.55)	11.57(1.61)	12.27(2.27)	76.75(12.25)
Male	12.74(3.96)	10.40(3.35)	11.06(2.86)	12.35(3.34)	11.21(2.18)	12.47(2.54)	70.23(16.44)
P value	<0.01	<0.05	<0.001	0.054	0.309	0.644	<0.05
**Marital status**							
Never married	14.01(2.74)	11.03(2.50)	12.61(2.38)	12.81(2.74)	11.06(1.38)	11.83(1.95)	73.36(11.23)
Currently married	15.22(4.82)	13.16(4.38)	13.00(2.50)	14.22(3.56)	12.89(2.20)	14.56(2.81)	83.04(18.96)
Divorced/widowed	10.80(3.16)	8.70(2.13)	8.84(2.15)	11.40(2.69)	10.64(2.25)	11.60(1.80)	61.98(12.26)
P value	<0.001	<0.001	<0.001	<0.01	<0.001	<0.001	<0.001
**Religion**							
Christianity	14.03(3.70)	11.26(3.11)	12.31(2.76)	12.82(3.00)	11.39(1.65)	12.17(2.26)	73.98(14.34)
Islam	15.17(3.28)	13.07(3.82)	13.00(2.06)	15.17(2.18)	12.67(2.05)	14.83(2.47)	83.90(14.63)
Other	11.62(3.05)	9.13(2.26)	10.28(2.78)	11.41(2.80)	10.53(2.20)	11.41(1.74)	64.39(12.02)
P value	<0.01	<0.001	<0.01	<0.001	<0.01	<0.001	<0.001
**Ethnicity**							
Akan	13.63(3.64)	10.93(3.09)	11.79(2.84)	12.53(2.99)	11.48(1.75)	12.30(2.26)	72.67(14.40)
Ewe	12.67(4.27)	10.40(2.50)	11.67(3.61)	12.67(3.28)	10.33(1.75)	10.67(0.50)	68.40(14.34)
Mole-Dagbani	13.96(3.65)	11.58(4.08)	12.56(2.47)	13.96(3.01)	11.36(2.56)	13.28(2.99)	76.70(17.13)
P value	0.666	0.571	0.465	0.115	0.241	<0.05	0.304
**Educational level**							
None	11.88(3.77)	10.75(3.10)	11.19(3.49)	12.06(3.75)	9.88(1.96)	12.31(2.44)	68.06(16.74)
Primary	11.86(4.00)	9.37(2.62)	11.43(2.62)	11.71(2.61)	11.21(2.05)	12.00(2.05)	67.59(14.37)
Secondary	13.52(3.54)	10.67(3.32)	11.50(3.03)	12.72(2.74)	11.04(1.47)	11.63(1.97)	71.10(13.57)
Tertiary	15.94(2.26)	12.93(2.84)	13.45(1.39)	14.13(3.12)	12.87(1.75)	14.03(2.65)	83.35(12.50)
P value	<0.001	<0.01	<0.01	<0.05	<0.001	<0.001	<0.001
**Residence**							
Rural	12.88(3.37)	9.60(1.42)	11.50(2.93)	11.25(2.64)	11.06(1.43)	11.63(1.53)	67.91(10.79)
Urban	13.81(3.73)	11.37(3.48)	12.04(2.80)	13.21(3.02)	11.45(2.05)	12.56(2.56)	74.44(15.63)
P value	0.264	<0.05	0.402	<0.01	0.383	0.089	0.055
**Employment**							
Unemployed	13.77(3.84)	10.45(3.29)	12(59(2.48)	12.75(3.08)	10.68(1.60)	11.82(1.90)	72.05(14.16)
Trader/business person	12.80(1.90)	11.36(2.31)	11.60(2.50)	13.00(1.73)	11.50(0.93)	12.20(1.21)	72.46(7.28)
Farmer/breeder	10.29(2.14)	8.80(0.00)	7.57(0.53)	8.57(0.53)	10.57(0.53)	11.43(0.53)	57.23(3.21)
Skilled worker	11.00(3.37)	9.95(2.85)	9.88(2.42)	13.13(2.55)	10.91(1.90)	11.50(2.31)	66.36(13.92)
Teacher	16.75(2.38)	13.60(3.34)	14.00(1.95)	13.75(3.72)	13.00(1.92)	14.25(2.99)	85.35(14.24)
Unskilled labourer	14.93(4.07)	12.46(4.30)	11.79(3.42)	13.86(3.32)	11.96(2.70)	13.86(3.74)	78.85(20.56)
Other	15.33(1.80)	11.73(1.74)	13.33(1.00)	13.00(3.00)	13.50(1.73)	13.33(2.18)	80.23(10.87)
P value	<0.001	<0.01	<0.001	<0.01	<0.001	<0.01	<0.001
**Years since diagnosis**							

(*Continued*)

Table 4. (Continued)

Variable	QoL domains						
	Physical	Psychological	Independence	Social	Environmental	Spiritual	Overall QoL
	Mean(S.D.)	Mean(S.D.)	Mean(S.D.)	Mean(S.D.)	Mean(S.D.)	Mean(S.D.)	Mean(S.D.)
**Age group**							
<10	14.25(3.23)	11.12(3.22)	12.35(2.43)	12.25(2.62)	11.33(1.69)	12.58(2.74)	73.87(13.63)
10–19	14.25(3.40)	11.79(3.07)	12.40(2.79)	13.81(2.91)	11.78(1.96)	12.51(2.43)	76.53(14.45)
20+	9.71(3.37)	7.81(2.05)	9.12(2.23)	10.35(2.87)	9.91(1.80)	11.41(0.94)	58.31(11.52)
P value	<0.001	<0.001	<0.001	<0.001	<0.01	0.205	<0.001
**Peer support group member**							
No	13.60(3.56)	10.72(3.05)	11.85(2.81)	12.64(2.83)	11.17(1.71)	12.21(2.39)	72.18(14.01)
Yes	13.76(4.25)	12.53(3.90)	12.38(2.92)	13.76(3.86)	12.41(2.65)	13.19(2.44)	78.03(18.80)
P value	0.856	<0.05	0.430	0.124	<0.01	0.091	0.104
**HIV/AIDS status disclosure**							
No	13.35(3.37)	10.90(2.92)	12.24(2.61)	12.85(3.03)	11.08(1.82)	12.04(2.09)	72.46(13.42)
Yes	14.02(4.05)	11.20(3.71)	11.52(3.07)	12.81(3.09)	11.78(2.06)	12.85(2.75)	74.17(17.03)
P value	0.316	0.615	0.164	0.943	0.049*	0.067	0.532

S.D = Standard Deviation

https://doi.org/10.1371/journal.pmen.0000060.t004

better QoL than those without any education. On the other hand, respondents who had tertiary education recorded lower QoL in the psychological domain than those who had no formal education, and this difference was statistically significant. Respondents who lived in urban areas had better QoL in all domains and overall QoL than those who lived in rural areas, and the difference was statistically significant. Although respondents who disclosed their status recorded better QoL overall than those who did not disclose their status, the difference was not statistically significant.

Discussions

We examined HIV/AIDS status disclosure and social support availability and their influence on quality of life among PLWHA in the Obuasi East District of the Ashanti Region, Ghana. We found that less than half of the HIV/AIDS clients had disclosed their status to someone such as a spouse, children, and nonfamily relations. Additionally, most of the clients had moderate quality of life across most of the six domains. We did not find a significant link between HIV/AIDS status disclosure and quality of life in any of the domains.

The finding that most HIV/AIDS clients did not disclose their status is congruent with many studies across the globe. Our status disclosure of 41.9% is consistent with the argument by Ssali et al. that HIV/AIDS status disclosure ranges from 24–91% [17] and is lower than the 49% reported by the WHO in the developing world [42]. Our finding could be attributed to the high levels of stigma and discrimination [43], fear of severed relationships, abuse [44], and feelings of shame in certain settings [45]. The finding suggests a need for interventions aimed at reducing stigma and discrimination to encourage more individuals to disclose their status, thus potentially improving overall QoL. Strategies for reducing stigma and discrimination among PLWHA may involve community education campaigns, workplace sensitivity training, and legal protections against discrimination based on HIV status.

We found in our study that HIV-positive patients had moderate QoL scores in all domains. This supports the argument that being on ART leads to better QoL, regardless of the duration of the ART [46]. It is therefore expected that most of the clients in this study would have better

Table 5. Effect of status disclosure on factors influencing quality of life among PLHIV.

Variable	QoL domains						Overall QoL
	Physical	Psychological	Independence	Social	Environmental	Spiritual	
	β(95%CI)	β(95%CI)	β(95%CI)	β(95%CI)	β(95%CI)	β(95%CI)	β(95%CI)
**Constant**	21.63(17.2, 26.1)***	20.75(16.9, 24.6)***	18.41(15.1, 21.7)***	19.99(15.9, 24.0)***	12.41(10.0, 14.8)***	15.61(12.5, 18.7)***	108.81(90.7, 126.9)***
**Age (in years)**	-0.23(-0.3, -0.1)***	-0.24(-0.3, -0.2)***	-0.18(-0.3, -0.1)***	-0.25(-0.3, -0.1)***	-0.07(-0.2, -0.1)*	-0.07(-0.1, 0.0)	-1.03(-1.5, -0.6)***
**Sex**							
Female	1	1	1	1	1	1	1
Male	-2.81(-4.4, -1.2)**	-2.82(-4.2, -1.4)***	-1.97(-3.1, -0.8)**	-2.23(-3.7, -0.8)**	-1.83(-2.7, -1.0)***	-1.47(-2.6, -0.4)*	-13.14(-19.6, -6.7)***
**Marital status**							
Never married	1	1	1	1	1	1	1
Currently married	0.28(-1.6, 2.1)	1.81(0.2, 3.4)*	-0.01(-1.4, 1.4)	1.43(-0.3, 3.1)	0.52(-0.5, 1.5)	2.29(1.0, 3.6)**	6.32(-1.3, 13.9)
Divorced/widowed	1.17(-1.4, 3.7)	2.41(0.2, 4.6)*	-0.88(-2.8, 1.0)	3.20(0.8, 5.6)**	0.80(-0.6, 2.2)	2.47(0.7, 4.3)**	9.17(-1.3, 19.7)
**Religion**							
Christianity	1	1	1	1	1	1	1
Islam	5.08(2.8, 7.3)***	4.73(2.8, 6.7)***	3.69(2.0, 5.4)***	5.12(3.1, 7.2)***	3.10(1.9, 4.3)***	3.86(2.3, 5.4)***	25.58(16.4, 34.8)***
Other	1.54(-0.6, 3.6)	-0.98(-2.8, 0.8)	1.87(0.3, 3.4)*	0.42(-1.5, 2.3)	0.57(-0.6, 1.7)	-0.26(-1.7, 1.2)	3.16(-5.4, 11.7)
**Ethnicity**							
Akan	1	1	1	1	1	1	1
Ewe	-2.19(-4.4, 0.0)	-2.18(-4.1, -0.3)*	-1.08(-2.7, 0.5)	-2.29(-4.3, -0.3)*	-0.82(-2.0, 0.4)	-1.84(-3.4, -0.3)*	-10.39(-19.3, -1.4)*
Mole-Dagbani	-2.07(-4.0, -0.1)*	-1.96(-3.6, -0.3)*	-0.88(-2.3, 0.5)	-1.47(-3.2, 0.3)	-1.25(-2.3, -0.2)*	-1.63(-3.0, -0.3)*	-9.26(-17.1, -1.4)*
**Educational level**							
None	1	1	1	1	1	1	1
Primary	-2.28(-4.7, 0.1)	-4.17(-6.2, -2.1)***	-1.00(-2.8, 0.8)	-1.65(-3.8, 0.5)	1.01(-0.3, 2.3)	-1.72(-3.4, -0.1)*	-9.80(-19.3, -1.4)*
Secondary	-1.49(-3.5, 0.5)	-4.17(-5.9, -2.4)***	-1.55(-3.0, -0.1)*	-2.12(-4.0, -0.3)*	0.20(-0.9, 1.3)	-2.60(-4.0, -1.2)***	-11.73(-20.0, -3.5)**
Tertiary	-0.12(-2.6, 2.3)	-3.14(-5.3, -1.0)**	-0.52(-2.3, 1.3)	-0.93(-3.2, 1.3)	1.35(0.0, 2.7)*	-1.26(-3.0, 0.5)	-4.63(-14.6, 5.4)
**Residence**							
Rural	1	1	1	1	1	1	1
Urban	2.11(0.8, 3.4)**	2.44(1.3, 3.6)***	1.07(0.1, 2.0)*	2.31(1.1, 3.5)***	0.77(0.1, 1.5)*.	1.24(0.3, 2.2)**	9.93(4.6, 15.2)***
**Employment**							
Unemployed	1	1		1	1	1	1
Trader/business person	-1.38(-3.4, 0.6)	-0.78(-2.5, 0.9)	-0.98(-2.4, 0.5)	-0.98(-2.8, 0.8)	-0.60(-1.7, 0.5)	-1.73(-3.1, -0.3)*	-6.46(-14.5, 1.6)
Farmer/breeder	-0.24(-3.3, 2.8)	3.01(0.4, 5.7)*	-1.34(-3.6, 0.9)	-1.75(-4.6, 1.0)	1.83(0.2, 3.5)*	-0.09(-2.3, 2.1)	1.42(-11.1, 13.9)
Skilled worker	-1.72(-3.6, 0.2)	0.93(-0.7, 2.6)	-1.63(-3.0, -0.2)*	1.12(-0.6, 2.8)	0.97(-0.1, 2.0)	-0.57(-1.9, 0.8)	-0.90(-8.6, 6.8)
Teacher	2.29(0.3, 4.3)*	2.33(0.6, 4.1)*	1.56(0.1, 3.0)*	0.56(-1.3, 2.4)	1.71(0.6, 2.8)**	1.15(-0.2, 2.6)	9.58(1.4, 17.8)*
Unskilled labourer	2.40(0.5, 4.2)*	3.01(1.4, 4.6)***	0.26(-1.1, 1.6)	1.41(-0.3, 3.1)	1.55(0.5, 2.6)**	1.32(0.0, 2.6)*	9.94(2.4, 17.5)*
Other	3.47(1.1, 5.9)**	3.41(1.3, 5.5)**	2.51(0.7, 4.3)**	0.92(-1.3, 3.1)	2.81(1.5, 4.1)***	0.01(-1.7, 1.7)	13.12(3.3, 22.9)**
**Years since diagnosis**	-0.05(-0.2, 0.1)	0.01(-0.1, 0.1)	0.04(-0.1, 0.1)	0.07(-0.1, 0.2)	0.00(-0.1, 0.1)	-0.03(-0.1, 0.1)	0.04(-0.4, 0.5)
**Peer support group member**							
No	1	1	1	1	1	1	1
Yes	-0.47(-1.9, 1.0)	0.74(-0.5, 2.0)	0.24(-0.8, 1.3)	-0.09(-1.4, 1.2)	0.72(-0.1, 1.5)	0.50(-0.5, 1.5)	1.64(-4.2, 7.5)
**HIV/AIDS status disclosure**							
No	1	1	1	1	1	1	1
Yes	1.17(-0.1, 2.5)	-0.25(-1.4, 0.9)	-0.02(-1.0, 0.9)	-0.10(-1.3, 1.1)	-0.01(-0.7, 0.7)	-0.12(-1.0, 0.8)	0.67(-4.6, 6.0)

(*Continued*)

Table 5. (Continued)

Variable	QoL domains						Overall QoL
	Physical	Psychological	Independence	Social	Environmental	Spiritual	
	β(95%CI)	β(95%CI)	β(95%CI)	β(95%CI)	β(95%CI)	β(95%CI)	β(95%CI)
**Adjusted R square**	0.515	0.540	0.556	0.412	0.487	0.444	0.518

*p<0.05
**p<0.01
***p<0.001; 1 = Reference category

https://doi.org/10.1371/journal.pmen.0000060.t005

QoL similar to those in other low-income countries [47–49]. This is because PLWHA attend routine ART clinics for their follow-up and receive counselling, care and treatment, which may have positively impacted all the domains of health-related QoL. Additional support or interventions may thus be needed to enhance QoL beyond medical treatment alone.

Our finding that disclosure of HIV/AIDS status was not a statistically significant predictor of quality of life is supported by a study by Agbeko et al. [50], who found that disclosure status of HIV/AIDS among PLWHA in the Ho municipality of Ghana was not statistically significant, although disclosure improved the overall QoL of PLWHA. Similarly, the findings of Chandra and colleagues that HIV serostatus disclosure was not significantly associated with QoL gives credence to our result [51]. Other studies, by contrast, established a statistically significant relationship between disclosure of HIV/AIDS status and quality of life [46, 52, 53]. Even though our finding was not statistically significant, the fact that disclosure improves the QoL of HIV/AIDS could be explained by the role of supportive relationships minimizing stigmatization and discrimination toward PLWHA, which consequently boosts the QoL of PLWHA. Supportive social networks have been shown to play a crucial role in buffering the negative impact of HIV/AIDS-related stigma and discrimination, thereby promoting psychological well-being and overall QoL among PLWHA. Additionally, policies and interventions aimed at reducing stigma and discrimination within communities could further amplify the positive effects of disclosure on QoL. Thus, while our study did not find a direct statistical link, the broader context suggests that disclosure may indeed contribute to improved QoL through its influence on social support and stigma reduction. Social support networks could be enhanced through establishing peer support groups, providing counselling services, and promoting community engagement through awareness-raising events and activities.

Furthermore, we discovered that age negatively predicted the overall quality of life of PLWHA to a statistically significant degree. In other words, as PLWHA grow older, their QoL becomes poorer. This finding is analogous to a Kazakhstan study that established that increasing age decreases the QoL of PLWHA [54]. In the same vein, Chinese and Pakistani studies found that age was negatively associated with QoL [55, 56]. Some authors have made contradictory findings, however. For instance, studies in Burkina Faso showed that older PLWHA were more likely to have higher QoL than younger PLWHA [49]. There seems to be a logical explanation for our finding on the relationship between age and QoL. As people grow older, they are more exposed to diseases that weaken their immune system, thus compounding their healthcare problems and undercutting their QoL. Likewise, mental health challenges are commonly associated with old age.

We also established that female PLWHA had significantly higher QoL in all domains than their male counterparts. This finding is partially supported by a study that showed that females had significantly higher QoL in the domain of physical well-being than males [57]. Other studies, however, suggest that being a male PLWHA is a positive predictor of QoL [49, 56, 58, 59].

Meanwhile, some researchers did not find a statistically significant relationship between QoL and the sex of PLWHA [13, 50]. There is no consensus in the literature on the influence of sex on the QoL of PLWHA. Both groups of researchers who found opposing relationships between sex and QoL of PLWHA rely on one explanation for their observations: gender roles. Gender roles resign females living with HIV/AIDS to discriminatory behaviour, which could hinder their QoL. Additionally, gender roles limit access to the financial resources of these females, which impedes their ability to fend for themselves and improve their QoL. The same gender roles hurt men living with HIV/AIDS, too, by putting financial stress on men—in their role as breadwinners of the family, which invariably affects their QoL.

Our study also pointed to the influence of geographic location on the QoL of PLWHA. Urban residency is a positive predictor of HIV/AIDS. Although studies are largely silent on the impact of geographical location on the QoL of PLWHA, the study by Shriharsha and Rentala [58] found that PLWHA in urban areas had significantly higher QoL, supporting our findings. Urban locations, especially in developing settings such as Ghana, provide an advantage regarding physical access to ART services, which empowers PLWHA and puts them in a better position to cope with the disease than their colleagues in rural areas. Improving access to healthcare services for PLWHA in different demographic groups may require initiatives such as mobile clinics for rural areas, transportation subsidies, and culturally sensitive healthcare provider training programs.

In addition to the geographical area, our findings revealed the influence of religion on the QoL of PLWHA. Belonging to the Islamic religion was a positive predictor of quality of life. The influence of religion on the QoL of PLWHA is an established concept [60, 61]. Religious bodies present an opportunity for social support for PLWHA. However, the particular reason for the advantage that the Islamic religion gives to PLWHA is unclear. Perhaps Islamic practices are more collegial and community-centred than Christian practices.

Moreover, the intersectionality of sociodemographic factors, including age, gender, geographic location, and religion, presents a complex interplay that influences the quality of life of PLWHA. In the Ghanaian context, cultural norms, access to resources, and community support systems may interact with these sociodemographic factors to shape the experiences and well-being of PLWHA. For example, older PLWHA in rural areas may face compounded challenges due to limited access to healthcare services and social support networks, leading to a significant impact on their QoL compared to younger counterparts in urban settings. Gender dynamics further complicate this scenario, as cultural norms and expectations may influence access to employment opportunities and financial resources differently for men and women living with HIV/AIDS. Additionally, geographic location plays a pivotal role; while urban areas typically offer better access to healthcare facilities and support services, factors such as stigma and discrimination may still persist, particularly affecting QoL among PLWHA in densely populated urban centres. Moreover, religious affiliation can influence social support structures and coping mechanisms, with religious communities often providing essential support networks for PLWHA. For instance, mosques and churches may serve as hubs for community gatherings and support groups, facilitating emotional support and enhancing QoL for PLWHA. Understanding these complex interactions is crucial for tailoring targeted interventions and policies to address the diverse needs of PLWHA across different sociodemographic groups. Exploring potential barriers or challenges to implementing these interventions could involve assessing cultural beliefs, addressing healthcare infrastructure limitations, and overcoming financial constraints for both individuals and healthcare systems. Collaborating with local community leaders, leveraging technology for remote healthcare delivery, and advocating for policy changes to support comprehensive HIV/AIDS care can help overcome these barriers.

Strengths and limitations

This study derives its strength from the use of a standard survey instrument in assessing the quality of life of PLWHA. The adoption of higher-level statistical analytical approaches (multiple linear regression) in this study enhances the accuracy of the findings and allows for a deeper understanding of the relationships between variables. Despite these strengths, several limitations must be acknowledged to contextualize the interpretation and generalizability of the findings. The relatively small sample size restricts the extent to which the results can be extrapolated to broader populations of PLWHA. Again, the quantitative nature of the study limits the robustness of the findings, as some important sociocultural factors that may also influence the quality of life of PLWHA can only be assessed qualitatively. Also, focusing on only one health facility in a municipality in Ghana raises concern about the generalizability of the findings to other health facilities within the municipality and the country as a whole. Variations in healthcare infrastructure, resources, and patient demographics across different facilities may impact the applicability of the findings to broader contexts. Finally, the reliance on self-reported data introduces the possibility of social desirability bias, where participants may provide responses that align with societal expectations rather than their true experiences. While efforts were made to mitigate this bias through confidentiality and anonymity, its potential influence on the study findings should be acknowledged.

Conclusions

The finding that less than half of the PLWHA disclosed their HIV/AIDS status implies that most of their sexual and nonsexual relations are at risk of infection. Additionally, this suggests that persistent stigma and discrimination may contribute to the reluctance of most PLWHA to disclose their status. This may affect the fight to reduce the infection and improve the quality of life for PLWHA.

Moreover, the finding that most PLWHA had moderate quality of life implies that ART services likely have a positive impact on the clients' well-being. A good quality of life suggests improvement in the overall health of HIV/AIDS patients, potentially reducing morbidity and mortality associated with the disease. Ghana's chances of attaining goal 3.3 of the Sustainable Development Goals, which seeks to end the HIV/AIDS epidemic and reduce mortality associated with the condition, could be increased by stakeholders through initiatives that target and limit the influence of negative predictors of the quality of life of PLWHA.

Based on the implications of these findings, we recommend that the Ghana AIDS Commission and the Ghana Health Service intensify efforts to reduce discrimination and stigmatization associated with HIV/AIDS. Furthermore, stakeholders in AIDS prevention in Ghana should employ innovative methods such as peer support to encourage status disclosure and provide education on the provision of social and emotional support for PLWHA.

Supporting information

S1 Data. Study data.
(SAV)

Author Contributions

Conceptualization: Farrukh Ishaque Saah, Abdul-Aziz Seidu, Hubert Amu, Luchet Engelbert Bain.

Data curation: Farrukh Ishaque Saah.

Formal analysis: Farrukh Ishaque Saah.

Funding acquisition: Farrukh Ishaque Saah, Abdul-Aziz Seidu.

Investigation: Farrukh Ishaque Saah, Kizito Aidam.

Methodology: Farrukh Ishaque Saah, Abdul-Aziz Seidu, Hubert Amu, Luchet Engelbert Bain.

Project administration: Farrukh Ishaque Saah.

Resources: Farrukh Ishaque Saah, Hubert Amu.

Software: Hubert Amu.

Supervision: Farrukh Ishaque Saah, Abdul-Aziz Seidu, Hubert Amu, Luchet Engelbert Bain.

Validation: Farrukh Ishaque Saah, Kizito Aidam, Abdul-Aziz Seidu, Hubert Amu, Luchet Engelbert Bain.

Visualization: Farrukh Ishaque Saah.

Writing – original draft: Farrukh Ishaque Saah, Kizito Aidam.

Writing – review & editing: Farrukh Ishaque Saah, Kizito Aidam, Abdul-Aziz Seidu, Hubert Amu, Luchet Engelbert Bain.

References

1. Osinde MO, Kakaire O, Kaye DK. Factors associated with disclosure of HIV serostatus to sexual partners of patients receiving HIV care in Kabale, Uganda. Int J Gynecol Obstet. 2012; 118(1):61–64. https://doi.org/10.1016/j.ijgo.2012.02.008 PMID: 22507263
2. Broyles LN, Luo R, Boeras D, Vojnov L. The risk of sexual transmission of HIV in individuals with low-level HIV viraemia: A systematic review. Lancet. 2023; 402(10400):464–471. https://doi.org/10.1016/S0140-6736(23)00877-2 PMID: 37490935
3. World Health Organization (WHO). WHO: Antiretroviral therapy. [Internet]. 2014. [cited 2019 Apr 20]. Available from: http://www.who.int/topics/antiretroviral_therapy/en/
4. PEPFAR. Global HIV/AIDS overview. [Internet]. 2023. [cited 2023 Sep 15]. Available from: https://www.hiv.gov/federal-response/pepfar-global-aids/global-hiv-aids-overview/
5. Eustace RW, Ilagan PR. HIV disclosure among HIV positive individuals: A concept analysis. J Adv Nurs. 2010; 66(9):2094–2103. https://doi.org/10.1111/j.1365-2648.2010.05354.x PMID: 20626492
6. Yokoyama K, Morimoto T, Ichihara-Takeda S, Yoshino J, Matsuyama K, Ikeda N. Relationship between self-disclosure to first acquaintances and subjective well-being in people with schizophrenia spectrum disorders living in the community. PLoS One. 2019; 14(10):e0223819. https://doi.org/10.1371/journal.pone.0223819 PMID: 31618271
7. Zhang R. The stress-buffering effect of self-disclosure on Facebook: An examination of stressful life events, social support, and mental health among college students. Comput Human Behav. 2017; 75:527–537.
8. Turan B, Hatcher AM, Weiser SD, Johnson MO, Rice WS, Turan JM. Framing mechanisms linking HIV-related stigma, adherence to treatment, and health outcomes. Am J Public Health. 2017; 107(6):863–869. https://doi.org/10.2105/AJPH.2017.303744 PMID: 28426316
9. Atuyambe LM, Ssegujja E, Ssali S, Tumwine C, Nekesa N, Nannungi A, et al. HIV/AIDS status disclosure increases support, behavioural change and, HIV prevention in the long term: a case for an Urban Clinic, Kampala, Uganda. BMC Health Serv Res. 2014; 14:276. https://doi.org/10.1186/1472-6963-14-276 PMID: 24950958
10. World Health Organization (WHO). Guidelines on HIV disclosure for children up to 12 years of age. Geneva: WHO Press; 2011.
11. Huang H. Examining the beneficial effects of individual's self-disclosure on the social network site. Comput Human Behav. 2016; 57:122–132.
12. Gari T, Habte D, Markos E. HIV positive status disclosure among women attending art clinic at Hawassa University Referral Hospital, South Ethiopia. East Afr J Public Health. 2010; 7(1):87–91. PMID: 21413581

13. Folasire OF, Irabor AE, Folasire AM. Quality of life of People living with HIV and AIDS attending the Antiretroviral Clinic, University College Hospital, Nigeria. Afr J Prim Health Care Fam Med. 2012; 4 (1):1–8.

14. Shushtari ZJ, Sajjadi H, Forouzan AS, Salimi Y, Dejman M. Disclosure of HIV Status and Social Support Among People Living With HIV. Iran Red Crescent Med J. 2014; 16(8):e11856. https://doi.org/10.5812/ircmj.11856 PMID: 25389470

15. Hogwood J, Campbell T, Butler S. I wish I could tell you but I can't: Adolescents with perinatally acquired HIV and their dilemmas around self-disclosure. Clin Child Psychol Psychiatry. 2013; 18(1):44–60. https://doi.org/10.1177/1359104511433195 PMID: 22287554

16. Stutterheim SE, Shiripinda I, Bos AER, Pryor JB, de Bruin M, Nellen JFJB, et al. HIV status disclosure among HIV-positive African and Afro-Caribbean people in the Netherlands. AIDS Care. 2011; 23 (2):195–205. https://doi.org/10.1080/09540121.2010.498873 PMID: 21259132

17. Ssali SN, Atuyambe L, Tumwine C, Segujja E, Nekesa N, Nannungi A, et al. Reasons for disclosure of HIV status by people living with HIV/AIDS and in HIV care in Uganda: An exploratory study. AIDS Patient Care STDs. 2010; 24(10):675–681. https://doi.org/10.1089/apc.2010.0062 PMID: 20863244

18. DeCarlo P, Ekstrand M. How does stigma affect HIV prevention and treatment? San Francisco: Center for AIDS Prevention Studies; 2016.

19. Ahsan Ullah AKM. HIV/AIDS-related stigma and discrimination: A study of health care providers in Bangladesh. J Int Assoc Physicians AIDS Care (Chic). 2011; 10(2):97–104. https://doi.org/10.1177/1545109710381926 PMID: 21278365

20. Wamoyi J, Renju J, Moshabela M, McLean E, Nyato D, Mbata D, et al. Understanding the relationship between couple dynamics and engagement with HIV care services: insights from a qualitative study in Eastern and Southern Africa. Sex Transm Infect. 2017; 93(Suppl 3). https://doi.org/10.1136/sextrans-2016-052976 PMID: 28736395

21. Bott S, Obermeyer CM. The social and gender context of HIV disclosure in sub-Saharan Africa: A review of policies and practices. SAHARA J. 2013; 10:S5–S16. https://doi.org/10.1080/02664763.2012.755319 PMID: 23808487

22. Greeff M, Phetlhu R, Makoae LN, Dlamini PS, Holzemer WL, Naidoo JR, et al. Disclosure of HIV status: experiences and perceptions of persons living with HIV/AIDS and nurses involved in their care in Africa. Qual Health Res. 2008; 18(3):311–324. https://doi.org/10.1177/1049732307311118 PMID: 18235155

23. Campbell C, Nair Y, Maimane S, Nicholson J. 'Dying Twice' A multi-level model of the roots of AIDS stigma in two South African communities. J Health Psychol. 2007; 12(3):403–416. https://doi.org/10.1177/1359105307076229 PMID: 17439992

24. Bonnington O, Wamoyi J, Ddaaki W, Bukenya D, Ondenge K, Skovdal M, et al. Changing forms of HIV-related stigma along the HIV care and treatment continuum in sub-Saharan Africa: a temporal analysis. Sex Transm Infect. 2017; 93(Suppl 3). https://doi.org/10.1136/sextrans-2016-052975 PMID: 28736394

25. Amo-Adjei J, Darteh EK. Drivers of young people's attitudes towards HIV/AIDS stigma and discrimination: evidence from Ghana. Afr J Reprod Health. 2013; 17(4):51–9. PMID: 24689316

26. Kalembo FW, Kendall GE, Ali M, Chimwaza AF. Socio-demographic, clinical, and psychosocial factors associated with primary caregivers' decisions regarding HIV disclosure to their child aged between 6 and 12 years living with HIV in Malawi. PLoS One. 2019; 14(1):e0210781. https://doi.org/10.1371/journal.pone.0210781 PMID: 30645639

27. Poku RA, Owusu AY, Mullen PD, Markham C, McCurdy SA. Considerations for purposeful HIV status disclosure among women living with HIV in Ghana. AIDS Care. 2017; 29(5):541–544. https://doi.org/10.1080/09540121.2016.1255711 PMID: 27838933

28. Britto C, Mehta K, Thomas R, Shet A. Prevalence and correlates of HIV disclosure among children and adolescents in low- and middle-income countries: a systematic review. J Dev Behav Pediatr. 2016; 37 (6):496. https://doi.org/10.1097/DBP.0000000000000303 PMID: 27262128

29. Ghana AIDS Commission. Country aids response progress report—Ghana. Reporting Period January 2012 –December 2013. Accra: Author; 2014.

30. Adam A, Fusheini A, Ayanore MA, Amuna N, Agbozo F, Kugbey N, et al. HIV stigma and status disclosure in three municipalities in Ghana. Ann Glob Health. 2021; 87(1):49. https://doi.org/10.5334/aogh.3120 PMID: 34164262

31. Ulasi CI, Preko PO, Baidoo JA, Bayard B, Ehiri JE, Jolly CM, et al. HIV/AIDS-related stigma in Kumasi, Ghana. Health Place. 2009; 15(1):255–262. https://doi.org/10.1016/j.healthplace.2008.05.006 PMID: 18632302

32. Chan KY, Rungpueng A, Reidpath DD. AIDS and the stigma of sexual promiscuity: Thai nurses' risk perceptions of occupational exposure to HIV. Cult Health Sex. 2009; 11(4):353–368. https://doi.org/10.1080/13691050802621161 PMID: 19263260

33. Bharat S. A systematic review of HIV/AIDS-related stigma and discrimination in India: current understanding and future needs. SAHARA J. 2011; 8(3):138–149. https://doi.org/10.1080/17290376.2011.9724996 PMID: 23237728
34. Megari K. Quality of life in chronic disease patients. Health Psychol Res. 2013; 1(3):e27. https://doi.org/10.4081/hpr.2013.e27 PMID: 26973912
35. World Health Organization (WHO) & Public Health Agency of Canada. Preventing chronic diseases: a vital investment. Geneva: World Health Organization; 2005.
36. United Nations Development Programme (UNDP). Vulnerability assessment of people living with HIV (PLHIV) in Lithuania: Final report. New York: UNDP; 2009.
37. Blackstock O. Curing stigma—the limits of antiretroviral access. N Engl J Med. 2005; 353(8):752. https://doi.org/10.1056/NEJMp058198 PMID: 16120853
38. Wouters E, van Loon F, van Rensburg D, Meulemans H. Community support and disclosure of HIV serostatus to family members by public-sector antiretroviral treatment patients in the Free State Province of South Africa. AIDS Patient Care STDs. 2009; 23(5):357–364. https://doi.org/10.1089/apc.2008.0201 PMID: 19327099
39. Ghana Districts. Obuasi East District Assembly. [Internet]. 2018. [cited 2019 Sep 18]. Available from: http://www.ghanadistricts.com/Home/District/228
40. Yamane T. Statistics: An introductory analysis. 2nd ed. New York: Harper and Row; 1967.
41. World Health Organization (WHO). WHOQOL-HIV BREF. Geneva: Author; 2002.
42. World Health Organization (WHO). The world health report 2003: Shaping the future. Geneva: World Health Organization; 2003.
43. Obermeyer CM, Baijal P, Pegurri E. Facilitating HIV disclosure across diverse settings: A review. Am J Public Health. 2011; 101(6):1011–1023. https://doi.org/10.2105/AJPH.2010.300102 PMID: 21493947
44. Obiri-Yeboah D, Amoako-Sakyi D, Baidoo I, Adu-Oppong A, Rheinländer T. The "Fears" of disclosing HIV status to sexual partners: a mixed methods study in a counseling setting in Ghana. AIDS Behav. 2016; 20(1):126–136. https://doi.org/10.1007/s10461-015-1022-1 PMID: 25711298
45. Asiedu GB, Myers-Bowman KS. Gender differences in the experiences of HIV/AIDS-related stigma: A qualitative study in Ghana. Health Care Women Int. 2014; 35(7–9):703–27. https://doi.org/10.1080/07399332.2014.895367 PMID: 24564483
46. Yaya I, Djalogue L, Patassi AA, Landoh DE, Assindo A, Nambiema A, et al. Health-related quality of life among people living with HIV/AIDS in Togo: individuals and contextual effects. BMC Res Notes. 2019; 12(1):140. https://doi.org/10.1186/s13104-019-4171-x PMID: 30876448
47. Davey DJ, Abrahams Z, Feinberg M, Prins M, Serrao C, Medeossi B, et al. Factors associated with recent unsuppressed viral load in HIV-1-infected patients in care on first-line antiretroviral therapy in South Africa. Int J STD AIDS. 2018; 29(6):603–610. https://doi.org/10.1177/0956462417748859 PMID: 29334886
48. Bakiono F, Guiguimdé PWL, Sanou M, Ouédraogo L, Robert A. Quality of life in persons living with HIV in Burkina Faso: a follow-up over 12 months. BMC Public Health. 2015; 15(1):1119. https://doi.org/10.1186/s12889-015-2444-4 PMID: 26563970
49. Bakiono F, Ouédraogo L, Sanou M, Samadoulougou S, Guiguimdé PWL, Kirakoya-Samadoulougou F, et al. Quality of life in people living with HIV: a cross-sectional study in Ouagadougou, Burkina Faso. Springerplus. 2014; 3(1):372. https://doi.org/10.1186/2193-1801-3-372 PMID: 25089255
50. Agbeko A, Owusu R, Alhassan Y, Letsa T, Axame WK, Ogum-Alangea D. HIV Status Disclosure and Quality of Life of People Living with HIV/AIDS in the Ho Municipality, Ghana. Adv Public Health. 2022; 2022.
51. Chandra PS, Deepthivarma S, Jairam KR, Thomas T. Relationship of psychological morbidity and quality of life to illness-related disclosure among HIV-infected persons. J Psychosom Res. 2003; 54:199–203. https://doi.org/10.1016/s0022-3999(02)00567-6 PMID: 12614829
52. Bunjoungmanee P, Chunloy K, Tangsathapornpong A, Khawcharoenporn T, Apisarnthanarak A. Quality of life assessment among patients living with HIV/AIDS at a tertiary care hospital in Thailand. Southeast Asian J Trop Med Public Health. 2014; 45:834–42. PMID: 25427351
53. Hasanah CI, Zaliha AR, Mahiran M. Factors influencing the quality of life in patients with HIV in Malaysia. Qual Life Res Int J Qual Life Asp Treat Care Rehabil. 2011; 20:91–100. https://doi.org/10.1007/s11136-010-9729-y PMID: 20737215
54. Zhakipbayeva BT, Nugmanova ZS, Tracy M, Birkhead GS, Akhmetova GM, DeHovitz J. Factors influencing the quality of life in persons living with human immunodeficiency virus infection in Almaty, Kazakhstan. Int J STD AIDS. 2019; 30(13):1318–1328. https://doi.org/10.1177/0956462419876484 PMID: 31726932

55. Liping M, Peng X, Haijiang L, Lahong J, Fan L. Quality of life of people living with HIV/AIDS: a cross-sectional study in Zhejiang Province, China. PLoS One. 2015; 10(8):e0135705. https://doi.org/10.1371/journal.pone.0135705 PMID: 26308088

56. Ahmed A, Saqlain M, Bashir N, Dujaili J, Hashmi F, Mazhar F, et al. Health-related quality of life and its predictors among adults living with HIV/AIDS and receiving antiretroviral therapy in Pakistan. Qual Life Res. 2021; 30:1653–64. https://doi.org/10.1007/s11136-021-02771-y PMID: 33582967

57. Jaquet A, Garanet F, Balestre E, Ekouevi DK, Azani JC, Bognounou R, et al. Antiretroviral treatment and quality of life in Africans living with HIV: 12-month follow-up in Burkina Faso. J Int AIDS Soc. 2013; 16(1):18867. https://doi.org/10.7448/IAS.16.1.18867 PMID: 24369739

58. Shriharsha C, Rentala S. Quality of life among people living with HIV/AIDS and its predictors: A cross-sectional study at ART center, Bagalkot, Karnataka. J Family Med Prim Care. 2019; 8(3):1011. https://doi.org/10.4103/jfmpc.jfmpc_411_18 PMID: 31041243

59. Nobre N, Pereira M, Roine RP, Sintonen H, Sutinen J. Factors associated with the quality of life of people living with HIV in Finland. AIDS Care. 2017; 29(8):1074–1078. https://doi.org/10.1080/09540121.2017.1281879 PMID: 28110552

60. Grill KB, Wang J, Cheng YI, Lyon ME. The role of religiousness and spirituality in health-related quality of life of persons living with HIV: A latent class analysis. Psychol Relig Spirit. 2020; 12(4):494. https://doi.org/10.1037/rel0000301 PMID: 33777311

61. Tsevat J. Spirituality/religion and quality of life in patients with HIV/AIDS. J Gen Intern Med. 2006; 21 (Suppl 5):S1.
